# Metabolic Fingerprint of PS3-Induced Resistance of Grapevine Leaves against *Plasmopara viticola* Revealed Differences in Elicitor-Triggered Defenses

**DOI:** 10.3389/fpls.2017.00101

**Published:** 2017-02-14

**Authors:** Marielle Adrian, Marianna Lucio, Chloé Roullier-Gall, Marie-Claire Héloir, Sophie Trouvelot, Xavier Daire, Basem Kanawati, Christelle Lemaître-Guillier, Benoît Poinssot, Régis Gougeon, Philippe Schmitt-Kopplin

**Affiliations:** ^1^Agroécologie, AgroSup Dijon, CNRS, INRA, Université Bourgogne Franche-ComtéDijon, France; ^2^Analytical BioGeoChemistry, Helmholtz Zentrum München, German Research Center for Environmental HealthNeuherberg, Germany; ^3^UMR PAM Université de Bourgogne/AgroSupDijon, Institut Universitaire de la Vigne et du Vin, Jules GuyotDijon, France; ^4^Chair of Analytical Food Chemistry, Technische Universität MünchenFreising-Weihenstephan, Germany

**Keywords:** induced resistance, elicitor, grapevine, downy mildew, metabolomics

## Abstract

Induction of plant resistance against pathogens by defense elicitors constitutes an attractive strategy to reduce the use of fungicides in crop protection. However, all elicitors do not systematically confer protection against pathogens. Elicitor-induced resistance (IR) thus merits to be further characterized in order to understand what makes an elicitor efficient. In this study, the oligosaccharidic defense elicitors H13 and PS3, respectively, ineffective and effective to trigger resistance of grapevine leaves against downy mildew, were used to compare their effect on the global leaf metabolism. Ultra high resolution mass spectrometry (FT-ICR-MS) analysis allowed us to obtain and compare the specific metabolic fingerprint induced by each elicitor and to characterize the associated metabolic pathways. Moreover, erythritol phosphate was identified as a putative marker of elicitor-IR.

## Introduction

Wine industry is of high economic, social and cultural value worldwide (Bisson et al., 2002). However, viticulture has to adapt in order to face major changes such as climate evolution and environmental constraints. A true challenge is currently the evolution towards production systems combining sustainability, economical viability, and more eco-friendly practices.

The majority of the grown grapevine cultivars (*Vitis vinifera* L. cvs) are susceptible to cryptogamic diseases such as downy mildew or powdery mildew. Repeated pesticide applications are generally used to ensure the expected yield and harvest quality. However, some fungicides are harmful for the environment and human health, and contribute to the selection of resistant strains of pathogens (Casida, 2009; Nanni et al., 2016). Besides organic farming or the development of resistant varieties by grape breeding, one strategy that is likely to allow the reduction of the use of fungicides is the induction of plant resistance to pathogens by defense elicitors (Walters et al., 2013).

Elicitors are compounds of natural origin (derived from plants or microbes) or synthetically generated (Walters et al., 2013). Their perception by plant cells activates a cascade of signaling events, including H_2_O_2_ production, that induces defense gene expression, leading to defense responses, i.e., the synthesis of Pathogenesis-related (PR-) proteins, the antimicrobial compounds phytoalexins, and cell-wall reinforcement (Garcia-Brugger et al., 2006). Phytohormones such as jasmonic acid (JA), salicylic acid, and ethylene also contribute to signaling (Shigenaga and Argueso, 2016). Defense activation thus triggers a global cell reprogramming towards defense responses (Wiesel et al., 2014) and therefore generates a cost that the plant has to fuel with primary metabolism (Bolton, 2009; Rojas et al., 2014), sometimes at the expense of growth (Huot et al., 2014).

Several compounds were reported as elicitors of grapevine defenses (Adrian et al., 2012; Delaunois et al., 2013). Among them, the β-glucans laminarin and its sulfated derivative PS3 (Ménard et al., 2004) have been studied in details as they induce resistance against *Plasmopara viticola* (Pv), the obligate biotrophic oomycete responsible of downy mildew (Aziz et al., 2003; Trouvelot et al., 2008; Gauthier et al., 2014). The perception and subsequent biological activity of oligosaccharides is highly dependent on their degree of polymerization (DP) and molecular pattern, i.e., acetylation, methylation, and/or sulfation (Trouvelot et al., 2014; Paris et al., 2015). As example, the short laminarin H13 (or Lam13, DP of 13) triggers the production of H_2_O_2_ in grapevine leaves without subsequent induced resistance (IR) against Pv (Allègre et al., 2009). Conversely, PS3 (DP of 25, degree of sulfation of 2.4) primes H_2_O_2_ production and induces protection against downy mildew (Trouvelot et al., 2008; Allègre et al., 2009; Gauthier et al., 2014). Such observations also show that plant defense activation is necessary, but not sufficient to induce resistance against pathogens. Numerous studies have reported new elicitors and mainly focused on the analysis of induced defense events and their regulation, and their efficiency to protect plants against diseases^1^ (Wiesel et al., 2014). However, to our knowledge, no comparative study using elicitors effective/ineffective to trigger IR was previously performed. What makes the difference between defense activation and IR thus remains unclear and merits to be investigated.

Global approaches have become powerful tools for diverse biological features. Metagenomic, transcriptomic, proteomic and metabolomic methods, now called “omics,” indeed provide useful large-scale data to understand biological systems (Buescher and Driggers, 2016). Metabolomic is suitable for different scales, from cell to organs. Among the different analytical systems available, the high sensitivity of mass spectrometry makes it a tool of choice for most of the plant metabolism analyses (Jorge et al., 2016). In plants, metabolomics approaches have been used for various studies (Jorge et al., 2016) including development, such as leaf senescence (Kim et al., 2016), and response to biotic or abiotic stresses, such as salt resistance (Janz et al., 2010). In grapevine, metabolomic was mainly used for variety characterization (Flamini et al., 2013, 2014) and to study berry evolution (Suzuki et al., 2015), impact of environmental conditions on berry, must and wines (Roullier-Gall et al., 2014a; Matus, 2016), and response to drought stress (Hochberg et al., 2013; Griesser et al., 2015; Shiratake and Suzuki, 2016) or pathogens (Batovska et al., 2009; Becker et al., 2013; Agudelo-Romero et al., 2015).

The objective of this study was to better characterize elicitor- IR of the grapevine leaves against downy mildew. The two β-glucan elicitors H13 and PS3, respectively, ineffective and highly effective to induce resistance against downy mildew (i.e., unable and able to limit the pathogen development and confer a protection against the disease, respectively), were chosen. Ultra high resolution mass spectrometry (FT-ICR-MS) was used to perform a global metabolomic analysis in order to (i) obtain and compare the metabolic fingerprint induced by each elicitor, (ii) characterize the metabolic fingerprint and pathways associated to elicitor-IR, and (iii) find putative markers of elicitor-IR.

## Materials and Methods

### Plant Material

The grapevine cultivar *V. vinifera* L. cv. Marselan (Cabernet sauvignon × Grenache), susceptible to *Plasmopara viticola*, was used for this study. Plants were obtained from herbaceous cuttings placed in individual pots (10 cm × 10 cm × 7 cm) containing a mixture of blond peat and perlite (3:2, v/v). They were grown in a glasshouse at a temperature of 24 and 18°C (day and night, respectively) with a photoperiod of 16 h of light and at a relative humidity (RH) of 70 ± 10 % until they developed six leaves. Plants were daily sub-irrigated with a fertilizer solution (NPK 10–10–10, Plantin, France).

### Elicitor Treatment

Two elicitors provided by Goëmar Laboratories were used for this study: H13 (or Lam13, here noted “H”), a short laminarin with a DP of 13 (Allègre et al., 2009) and the sulfated laminarin PS3 (here noted “P”; Ménard et al., 2004; Trouvelot et al., 2008).

**FIGURE 1:**
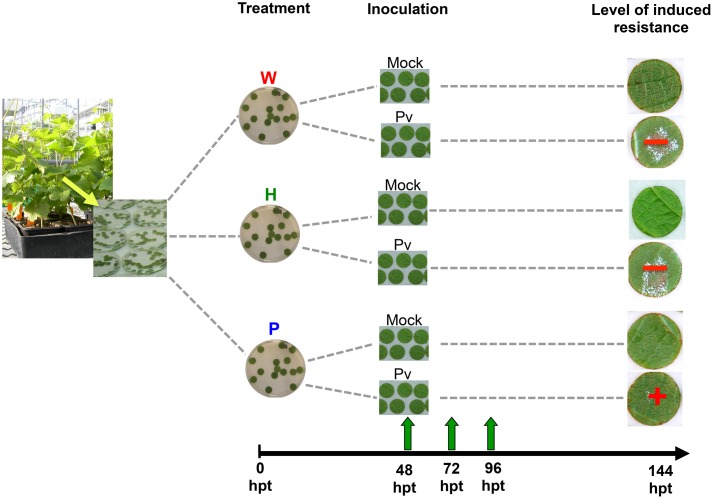
**Outline of the experiment.** Disks were punched out from leaves of *Vitis vinifera* L. cv. Marselan herbaceous plantlets and treated by distilled H_2_O (W, as control), H or P elicitor solutions (2.5 g.l^-1^). At 48 hpt, they were rinsed in distilled water and deposited on a moist paper filter before mock- or *P. viticola* inoculation (suspension of 10^4^ sporangia per milliliter of water). Thirty disks were prepared per condition, from the third leaves (under the apex) of three plants (*n* = 30). Disks were collected at 48, 72, and 96 hpt for analysis. Other ones were let until sporulation (144 hpt).

Leaf disks (14 mm diameter) were punched from leaves (the third below the apex) of grapevine plants grown in greenhouses as described above. They were washed in distilled water and placed at the surface (upper side above) of distilled H_2_O (W, as control), H or P solutions in water (2.5 g.l^-1^) in sterile Petri dishes (90 mm diameter) for 48 h (**Figure 1**). Afterwards, they were removed, rinsed in distilled water and deposited on a moist filter paper in a plastic box at room temperature before mock- or *Pv* inoculation. All disks were deposited on the same filter paper in the same plastic box to allow further tight comparison between treatments. Thirty disks were prepared per condition, from the third leaves of three plants (*n* = 30).

### *Plasmopara viticola* Infection

Sporangia were collected from sporulating leaves of Marselan herbaceous cuttings as previously described (Trouvelot et al., 2008) and their concentration was adjusted to 10^4^ sporangia per milliliter using an hemacytometer. At 48 hours after the beginning of the treatment (48 hpt), leaf disks were inoculated by a droplet (70 μl) of either the Pv sporangia suspension or water (mock) and incubated at room temperature (**Figure 1**).

### Sample Preparation

At 48 hpt (i.e., just before Pv- or mock- inoculation), 72 and 96 hpt, the disks were reduced to 12 mm in diameter using a cork borer (to eliminate the outlying area where defense responses to injury occured) and grind in liquid nitrogen. Samples were then stored at –80°C until extraction and analysis. Three disks were frozen separately per condition. The remaining disks were kept until 7 days post inoculation (dpi) in the same conditions in order to quantify the level of sporulation as previously described (Kim Khiook et al., 2013; **Figure 1**).

### FT-ICR-MS Analysis

Prior to analysis, 15 mg of each sample were mixed with 1 ml methanol (LC-MS grade, Fluka Analytical; Sigma-Aldrich, St. Louis, USA) and sonicated for 30 min. After centrifugation (25,000 *g*, 10 min, room temperature), the supernatant was collected and re-diluted in methanol (1/50 v/v). Ultra high-resolution mass spectra were acquired using an Ion Cyclotron Resonance Fourier Transform Mass Spectrometer (FT-ICR-MS) (solariX, BrukerDaltonics GmbH, Bremen, Germany) equipped with a 12 Tesla superconducting magnet (Magnex Scientific Inc., Yarnton, GB) and an APOLO II ESI source (Bruker Daltonics GmbH, Bremen, Germany) operated in the negative ionization mode. Samples were introduced into the micro electrospray source at a flow rate of 120 μl.h^-1^. The MS was externally calibrated on clusters of arginine (10 mg.l^-1^ in methanol). Spectra were acquired with a time domain of four mega words over a mass range of 100 to 1,000 Da and 300 scans were accumulated per sample.

### Data Analysis

Spectra were internally recalibrated using a list of ubiquitous fatty acids and recurrent wine compounds, allowing mass accuracies of 0.1 ppm (Gougeon et al., 2009). The *m/z* peaks with a signal-to-noise ratio (S/N) of 4 and higher were exported to peak lists. Exact masses were then subjected to Netcalc algorithm and in-house software tool to obtain chemical formulas (Tziotis et al., 2011) validated by setting sensible chemical constraints (n rule; O/C ratio ≤ 1; H/C ratio ≤ 2n+2; element counts: C ≤ 100, H ≤ 200, O ≤ 80, N ≤ 3, S ≤ 3, and P ≤ 1). In order to facilitate the interpretation of elemental formulas attributed to *m/z* values, formulas were next represented using two-dimensional van Krevelen (VK) diagrams. These diagrams display the hydrogen/carbon (H/C) versus oxygen/carbon (O/C) ratios of annotated elemental formulas and provide a commonly used qualitative description of the molecular complexity of biological samples (Tziotis et al., 2011; Roullier-Gall et al., 2014b). These plots provide additional information to function or biochemical pathways, through the representation of areas covering the various metabolite classes of samples. As such, they allow the qualitative comparison between series of related samples, in terms of their chemical complexity. The m/z distribution according to their elemental compositions (CHO, CHOS, CHON, CHONS, CHOP, CHONP CHONSP) was also represented. Mass annotations were obtained by KEGG, HMDB and LipidMaps databases queries. The metabolic pathways associated to selected masses were obtained by KEGG query with *Vitis vinifera* (vvi) as organism using the web server MassTRIX. Network analysis was performed from the complete sample set data as previously described (Roullier-Gall et al., 2014b). Nodes represent *m/z* values (metabolite candidates) and edges represent chemical reactions.

OPLS-DA (Orthogonal Projections to Latent Structures-Discriminant Analysis) was applied to datasets in order to discriminate between different sample groups. The overfitting was checked by the p-value calculated with the CV-ANOVA (Cross Validation ANOVA). From the classification models, the *m/z* values with the highest regression coefficient were extrapolated in different lists, as representative masses for the different clusters (called “top *m/z*”). These analysis were performed with SIMCA-P+13.0.3 (Umetrics, Umea, Sweden). Moreover for the analysis of the 96i and 72i datasets, it was shown a great improvement of the classification models applying before them the Relief algorithm. This chooses the features that can be most distinguished between classes (Package FSelector, Rstudio Inc., Version 0.99.896).

## Results

### W48, H48, and P48 Samples Had Specific Metabolic Fingerprints

**FIGURE 2:**
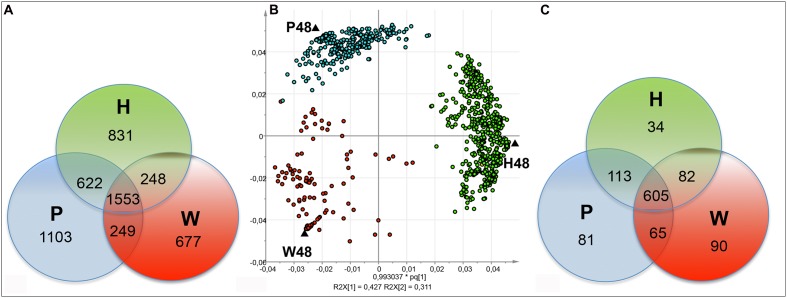
**Discrimination of FT-ICR-MS data of the 48 hpt samples.** Venn diagrams showing the distribution of the total **(A)** and top **(C)** *m/z* obtained by (–) FT-ICR-MS analysis of W48, H48, and P48 samples. Score plot **(B)** for the OPLS-DA analysis showing the discrimination of the W48, H48, and P48 samples. W48, H48, and P48 correspond to methanol extracts of grapevine leaf disks treated with H_2_O (W, as control), H (H13), or P (PS3) elicitor solutions (2.5 g.l^-1^) for 48 h. Top *m/z* correspond to *m/z* with the highest regression coefficient value (VIP ≥ 1).

The FT-ICR-MS analysis of all the samples provided a total of 8,067 *m/z*. The corresponding chemical composition histogram and VK diagram highlighted a wide chemical diversity, with a significant contribution of CHO elemental formulas (**Supplementary Figure S1**). A total of 5,283 *m/z* was present in the 48 hpt samples. Among them, 677, 831, and 1,103 ones were specific to W48, H48, and P48, respectively, whereas 1,553 ones were common to the three series (**Figure 2A**). The corresponding VK diagrams and chemical composition histograms obtained from W48, H48, and P48 did not show notable differences between these three sample sets (**Supplementary Figure S2**).

**FIGURE 3:**
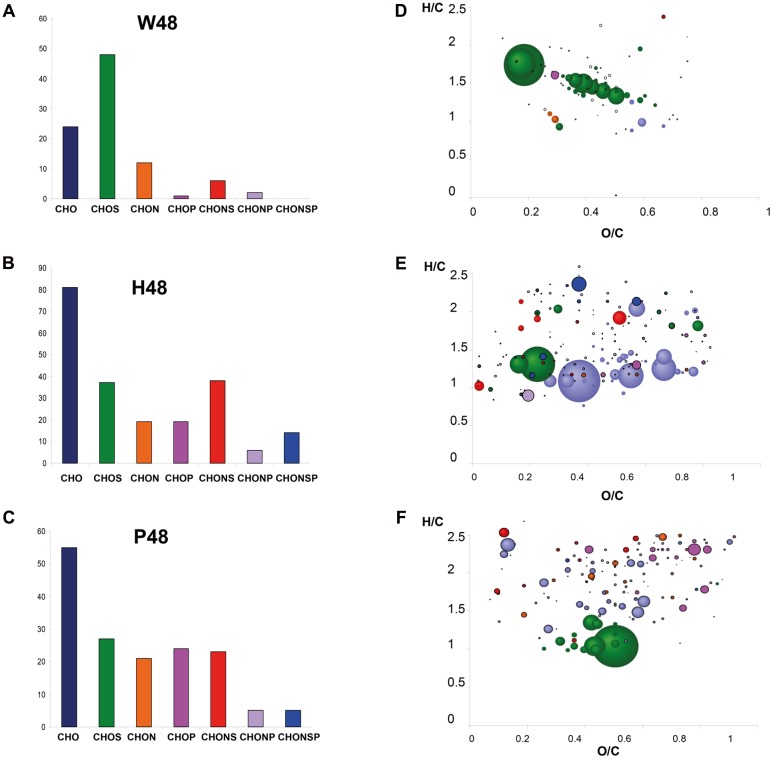
**Detailed visualization of the 48 hpt samples analyzed by FT-ICR-MS.** Chemical histograms show the distribution (number) of the elemental formulas attributed to the top *m/z* of W48 **(A)**, H48 **(B)**, and P48 **(C)** samples analyzed by (–) FT-ICR-MS, according to their elemental composition (CHO, CHOS, CHON, CHONS, CHOP, CHONP, or CHONSP). Their corresponding Van Krevelen diagrams **(D–F)** represent these formulas onto two axes according H/C and O/C atomic ratios. Dots are colored according to their elemental composition (CHO, CHOS, CHON, CHONS, CHOP, CHONP, CHONSP) and sized according to their relative intensity in mass spectra. W48, H48, and P48 correspond to methanol extracts of grapevine leaf disks treated with H_2_O (W, as control), H (H13), or P (PS3) elicitor solutions (2.5 g.l^-1^) for 48 h. Top *m/z* correspond to *m/z* with the highest regression coefficient value (VIP ≥ 1).

Orthogonal Projections to Latent Structures-Discriminant Analysis was next applied to discriminate W48, H48, and P48 samples (**Figure 2B**). W48 could be separated from P48 and H48 (*p*-value = 0.02), H48 from P48 and W48 (*p*-value = 0.05), and P48 from H48 and W48 (*p*-value = 0.06) (**Table 1**). Masses with the highest regression coefficient values in each sample group (specific and common masses with a variable importance in projection (VIP) value >1; thereafter called “top *m/z*”) were next used for comparison. A total of 1,070 top *m/z* were isolated for W48, H48, and P48 (**Table 2**). Among them, 90, 34 and 81 were specific to W48, H48, and P48, respectively (**Figure 2C**), representing about 50, 6, and 26% of their total top *m/z* number. The discrimination between the three sample sets was further characterized using the chemical histogram and VK diagram built from the elemental formulas attributed to their respective top *m/z* list (**Figure 3**). W48 chemical histogram was highly different from H48 and P48 ones, with a higher number of CHOS elemental formulas and a lower number of CHO, CHOP, CHONS, CHON, CHONP, and CHONSP ones (**Figures 3A–C**). VK diagrams also showed noteworthy differences in the nature and the number of formulas assigned to masses between the three sample groups (**Figures 3D–F**), leading to specific fingerprints. W48 had a greater diversity in CHOS formulas. H48 presented a wide chemical diversity, with important CHO formulas. P48 was chemically less diversified than H48 and showed a CHOS specificity.

**Table 1:** Significance of the sample discrimination performed by OPLS-DA analysis.

Model	Type	CV-Anova	*R*Y (cum)	*Q* (cum)
**48**
H vs. (P, W)		0.05	0.94	0.62
P vs. (H, W)	OPLS-DA	0.06	0.82	0.6
W vs. (H, P)		0.02	0.87	0.69
**72i**
H vs. (P, W)		0.03	0,97	0,881
P vs. (H, W)	OPLS-DA	0.02	0,998	0,963
W vs. (H, P)		0.02	0,886	0,689
**96i**
H vs. (P,W)		0.04	0,977	0,866
P vs. (H, W)	OPLS-DA	0.02	1	0,991
W vs. (H, P)		0.04	0,964	0,865

*W, H, and P correspond to methanol extracts of grapevine leaf disks treated with H_*2*_O (W, as control), H (H13), or P (PS3) elicitor solutions (2.5 g.l^-*1*^) for 48 h. *R*^*2*^Y and *Q*^*2*^ are two values calculated after the cross validation of the model; they represent the measurements of the goodness of the fit and the predictive quality of the model*.

**Table 2:** Number of top m/z determined for each treatment and sampling time.

	W	H	P	TOTAL
48	181	580	309	1070
72i	114	92	159	365
96i	36	79	146	261

*W, H, and P correspond to methanol extracts of grapevine leaf disks treated with H_*2*_O (W, as control), H (H13) or P (PS3) elicitor solutions (2.5 g.l^-*1*^) for 48 h*.

**FIGURE 4:**
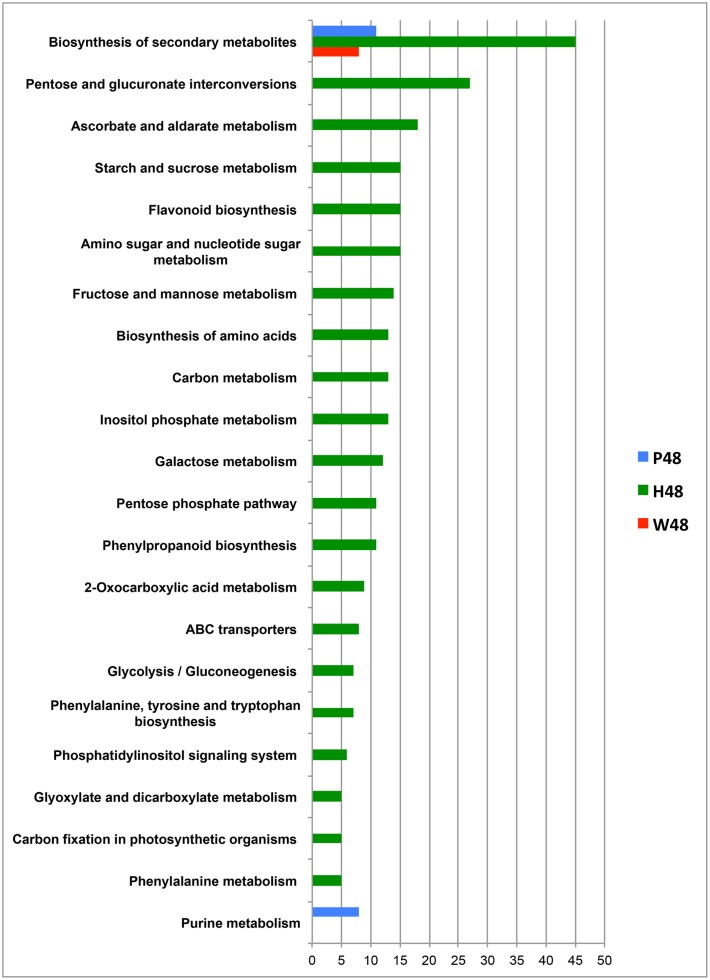
**Metabolic pathways associated to the top *m/z* identified in the 48 hpt samples.** The metabolic pathways correspond to the top *m/z* of W48 (red), H48 (green), and P48 (blue) samples. These pathways were obtained after KEGG query with the MassTRIX interface using *Vitis vinifera* organism (vvi). The x axis corresponds to the number of annotated formulas obtained for each pathway. Only pathways including at least five annotated formulas are presented. W48, H48, and P48 correspond to methanol extracts of grapevine leaf disks treated with H_2_O (W, as control), H (H13), or P (PS3) elicitor solutions (2.5 g.l^-1^) for 48 h. Top *m/z* correspond to *m/z* with the highest regression coefficient value (VIP ≥ 1).

Query of KEGG databasis (*Vitis vinifera* organism) with the MassTRIX interface allowed the annotation of top *m/z* for W48, H48, and P48, respectively, and their assignment to metabolic pathways (**Figure 4**). Few pathways were associated to W48 and P48 (**Figure 4**). “Biosynthesis of secondary metabolites” and “Purine metabolism” pathways were attributed to P48 top *m/z*. Among P48 annotated compounds were AMP, ADP, ATP, UTP, tryptophan, palmitic acid, oleic acid, JA, abscisic acid, traumatic acid, and syringin (**Figure 4**, **Supplementary Table S1**). H48 showed the highest diversity of pathways, covering “Biosynthesis of secondary metabolites”, “Pentose and glucuronate interconversions”, “Ascorbate and aldarate metabolism”, “Starch and sucrose metabolism”, “Amino sugar and nucleotide sugar metabolism”, and “Flavonoid biosynthesis” (**Figure 4**). These pathways included compounds annotated as ribose; fructose 6P, fructose 1,6 diP, trehalose 6P, glucosamine 6P, glyceraldehyde 3P, gluconic acid, ascorbic acid, the oxidized and reduced forms of glutathion, quercetin 3-*O*-glucoside, polydatine and 𝜀-viniferin (**Supplementary Table S1**). “Biosynthesis of secondary metabolites” was the unique pathway common to W48, H48, P48 top *m/z* (for a number of attribution exceeding 5) whereas “Purine metabolism” was specific to P48 (**Figure 4**).

### W, H, and P Fingerprints Evolved with *P. viticola* Infection and Time But Kept Specificity

At 48 hpt, leaf disks were mock- or *P. viticola* inoculated (thereafter noted “ni” and “i”, respectively) and collected at 72 and 96 hpt (i.e., 24 and 48 hpi, respectively; **Figure 1**). Relief algorithm combined with OPLS-DA analysis allowed the separation of W72i vs. P72i and H72i, H72i vs. P72i and W72i, and P72i vs. H72i and W72i with a significant *p*-value < 0.05 (**Table 1**). W96i, H96i, and P96i were similarly separated with a significant *p*-value < 0.05 (**Table 1**).

A total of 2,102 *m/z* was recorded for the 72i sample extracts. Among them, 83, 30, and 83 were specific to W72i, H72i, and P72i, respectively, whereas 402 were common to the three groups (**Supplementary Figure S3A**). As previously described for the 48 sample sets, the top *m/z* were identified and used for comparison. A total of 365 ones were obtained (**Table 2**), among with 70, 25, and 58 were specific to W72i, H72i, and P72i (**Supplementary Figure S3B**), i.e., 61, 27, and 36% of their total top *m/z*. The corresponding chemical composition and VK diagrams highlighted differences between W72i, H72i, and P72i (**Supplementary Figures S4A–C**). Compared to W72i and H72i, P72i metabolic fingerprint was characterized by a low level of CHO and CHOP formulas, and a high level of CHOS ones (**Supplementary Figures 4A–C**). The other formulas were few represented and distributed similarly to H72i ones. Compared to W72i, H72i had less CHOS, CHONP, and CHONSP formulas, and more CHOP ones (**Supplementary Figures S4A,B**). VK diagrams highlighted specific and highly different metabolic fingerprints for each sample set (**Supplementary Figures S4A–C**).

**FIGURE 5:**
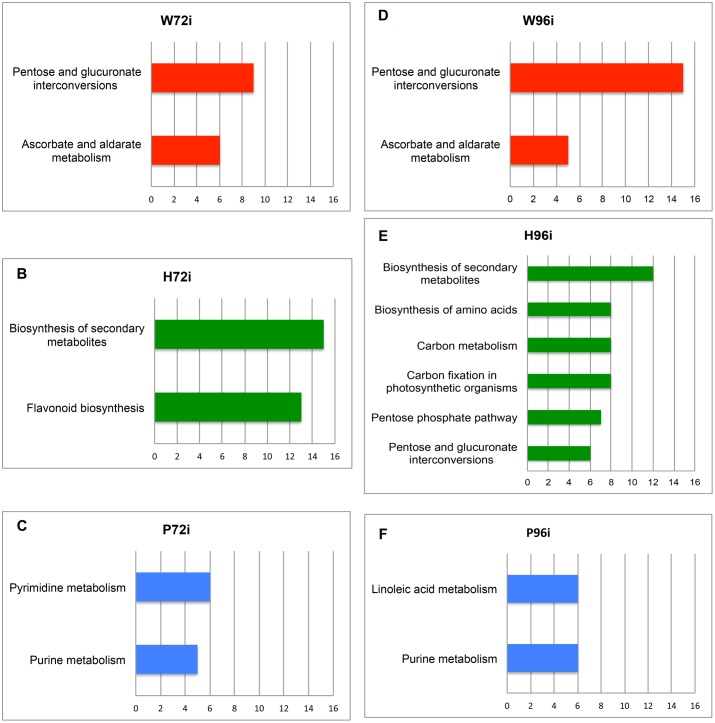
**Metabolic pathways associated to the top *m/z* identified in the 72i and 96i samples.** The metabolic pathways correspond to the top *m/z* of W72i **(A)**, H72i **(B)**, P72i **(C)**, W96i **(D)**, H96i **(E)**, and P96i **(F)** samples. These pathways were obtained after KEGG query with the MassTRIX interface using *Vitis vinifera* organism (vvi). The x axis correspond to the number of annotated formulas obtained for each pathway. Only pathways including at least five annotated formulas are presented. W72i/96i, H72i/96i, and P72i /96i correspond to methanol extracts of grapevine leaf disks treated with distilled H_2_O (W, as control), H (H13), or P (PS3) elicitor solutions (2.5 g.l^-1^) for 48 h and collected 72 and 96 h after *P. viticola* inoculation. Top *m/z* correspond to *m/z* with the highest regression coefficient value (VIP ≥ 1).

Query of KEGG databasis with the MassTRIX interface showed that “Pentose and glucuronate interconversions” and “Ascorbate and aldarate metabolism” were the main pathways associated to W72i top *m/z* (**Figure 5A**). “Biosynthesis of secondary metabolites” and “Flavonoid metabolism” were preferentially associated to H72i (**Figure 5B**) whereas the main pathways in P72i samples were “Purine metabolism” and “Pyrimidine metabolism” (**Figure 5C**). Among annotated masses were ADP, ATP, UMP, UDP for P72i and quercetin 7-*O*-glucoside, leucocyanidin, leucodelphinidin, 4-coumaroylshikimate, and ampelopsin for H72i (**Supplementary Table S2**).

A total of 1,808 *m/z* was recorded for the 96i sample extracts. Among them, 8, 25, and 153 were specific to W96i, H96i, and P96i, respectively, whereas 1,093 were common to the three series (**Supplementary Figure S3C**). A total of 261 top *m/z* were isolated (**Table 2**), among which 8, 25, and 49 were specific to W96i, H96i, and P96i, respectively (i.e., 22, 32, and 34% of their total top *m/z*) (**Supplementary Figure S3D**). P96i fingerprint was characterized by higher numbers of CHO, CHOS, and CHON formulas than W96i and H96i (**Supplementary Figures S4D–F**). The other chemical compositions were slightly present (CHOP, CHONS, CHONSP) or absent (CHONP). H96i displayed a majority of CHO and, to a lesser extent, of CHONSP formulas whereas all the other ones were meaningless. As few top *m/z* were obtained for W96i, rare or no formulas were found in the different chemical compositions. Query of KEGG databasis with the MassTRIX interface showed that the main pathways for W96i were “pentose and glucuronate interconversions”, and “ascorbate and aldarate metabolism” (**Figure 5D**), as for W72i (**Figure 5A**). For H96i, the main pathways were more diversified and mainly encompassed “biosynthesis of secondary metabolites “carbon metabolism”, “carbon fixation in photosynthetic organisms”, and “biosynthesis of amino acids”, “pentose phosphate pathways”, and “pentose and glucuronate interconversions” (**Figure 5E**). ”Linoleic acid metabolism” and “purine metabolism” were the main pathways for P96i (**Figure 5F**). Among the annotated masses were ADP, tetradecanoic acid, palmitoleic acid and hexadecanoic acid for P96i, d-ribose 5-P, d-ribose 5-diP, sedoheptulose-1,7-diP for H96i (**Supplementary Table S2**).

### Erythritol Phosphate: A Putative Marker of PS3-IR

**FIGURE 6:**
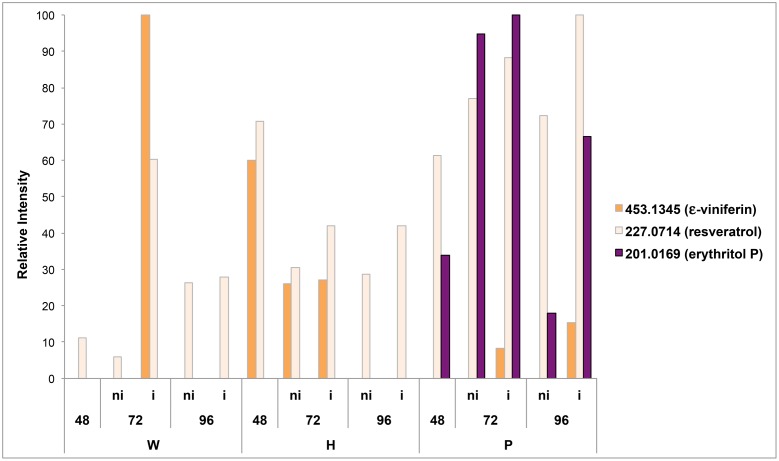
**Occurrence of resveratrol, 𝜀-viniferin and erythritol P in the different samples.** The signal intensities of putative resveratrol (*m/z* = 227, 0714), 𝜀-viniferin (*m/z* = 453,1345) and erythritol-P (*m/z* = 201.0169) are expressed in relative intensity. W, H, and P correspond to methanol extracts of grapevine leaf disks treated with distilled H_2_O (W, as control), H (H13), or P (PS3) elicitor solutions (2.5 g.l^-1^) for 48 h before *P. viticola* (i) or mock (ni) inoculation. Disks were collected at 48, 72, and 96 hpt.

Two metabolites previously reported as markers of grapevine defenses, resveratrol and 𝜀-viniferin, were first searched in the data files. Putative resveratrol (*m/z* 227.0714) was present in all samples, and transiently accumulated in response to elicitor treatment and infection (**Figure 6**). Putative 𝜀-viniferin (*m/z* 453.1345) was only induced in response to H treatment at 48 and 72 hpt, and in response to *P. viticola* infection at 72 hpt (for W and P treated leaves) and 96 hpt (for P treated leaves) (**Figure 6**).

**FIGURE 7:**
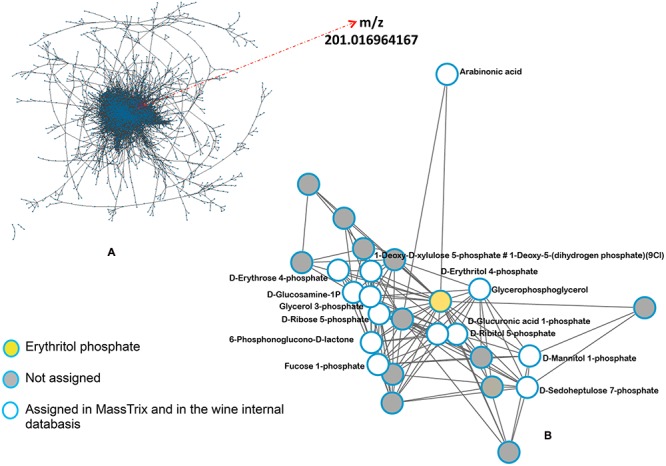
**Network analysis of the complete *m/z* data set.** The network was based on a list of possible transformations. **(A)** Network built from all *m/z* data sets and **(B)** focus on erythritol phosphate related network.

In order to find a putative marker of elicitor-IR, annotated top *m/z* specific to Pi series and present in both P72i and P96i were next investigated. Among the 58, and 49 top *m/z* specific to P72i and P96i, only 5 and 8 could be annotated, respectively. A single one, designed as erythritol phosphate (*m/z* = 201.0169), was common to both sample sets. The assessment of the presence of this compound in all samples confirmed that it specifically accumulated in P treated leaves (**Figure 6**). A network analysis based on a series of biochemical transformations (Roullier-Gall et al., 2014b) was performed to obtain an additional overview of the global metabolism of grapevine leaves (**Figure 7A**). A focus on erythritol phosphate *m/z* (**Figure 7B**) highlighted correlated elemental formulas. About half of them could be annotated with Masstrix and home made database. A large number of them was annotated to pentose phosphate pathway (**Supplementary Table S3**).

## Discussion

In order to better characterize elicitor-IR, a non-targeted analysis was performed to obtain a metabolite fingerprint associated to elicitor-IR and also to identify putative markers of elicitor-IR. FT-ICR-MS analyses were performed from methanolic extracts of leaf samples and in the negative mode. This method of extraction allows access to a wide number of *m/z* (Maia et al., 2016).

Supervised OPLS-DA analyses allowed the discrimination of the different sample series. The first sampling time used for comparison was 48 hpt, just before inoculation. This time was long enough to allow the accumulation of defense compounds (Trouvelot et al., 2008; Gauthier et al., 2014). W48, H48, and P48 sample series could be discriminated, indicating that grapevine leaf metabolism was differently modulated in response to these treatments. W48 had few top *m/z* with a dominance of CHOS containing metabolites. The presence of sulfur in these formulas was confirmed by highly exact mass measurements, including isotopic pattern analyses. However, it must be born in mind that elemental formulas do not represent all of the detected m/z features, but only those top features, which weigh for the discrimination presented. The significance of this sulfur-containing signature certainly merits to be investigated in future experiments. H48 had the highest number of top *m/z*, indicating that a large number of chemically diverse metabolites contributed to H48 series discrimination. This was confirmed by the diversity of the activated metabolic pathways and suggested a global activation of the cell metabolism. The activation of carbohydrate metabolism suggested that energy may be recruited to fuel defense reactions, as previously reported (Bolton, 2009; Rojas et al., 2014). H48 metabolic fingerprint also involved “Secondary metabolites” and “Flavonoid biosynthesis”, indicating that the plant is in a defensive status. Despite the *m/z* corresponding to the grapevine phytoalexin resveratrol (*m/z* = 227,0714046) was significantly present in H48 and P48 samples series (**Figure 6**), it was not identified among the top *m/z*. Conversely, its glycosylated and dimer derivatives piceid (or polydatine) and 𝜀-viniferin, respectively, were listed among the annotated top *m/z* attributed to H48 samples (**Supplementary Table S1**).

As for W48, a limited number of metabolic pathways was associated to P48 top *m/z*. As reminded above, PS3 is known to act by priming and little is known about its effects in plants before pathogen infection. Gauthier et al. (2014) have previously reported transcriptional changes in response to PS3 treatment but this is the first time that a direct effect of a priming elicitor is observed at the metabolome level. P48 metabolic response involved “purine metabolism”, with AMP, ADP, ATP, UTP annotations, suggesting the constitution of a pool of nucleotides and the mobilization of energy sources and/or cofactors for enzyme activities. Traumatic acid (or *trans*-2-dodeceno-1.12-dicarboxylic acid) and its precursor linolenic acid were among the annotated top *m/z*. Despite traumatic acid remains little studied, in plant, it is known to be involved in response to injury by stimulation of cell division (Zimmerman and Coudron, 1979). The production of traumatic acid and other 12C derivatives of the hydroperoxide lyase pathway was also recently studied in *Nicotiana attenuata* leaves in response to wounding (Kallenbach et al., 2011). Traumatic acid could also play an important role in plant adaptation to environmental stresses, as reported by Pietryczuk et al. (2014) for *Chlorella vulgaris*. It would be interesting to further analyze its putative role in elicitor-IR. Other compounds such as JA and abscisic acid were also among the annotated top *m/z*, suggesting that the alpha-linolenic acid cascade (Rojas et al., 2014) was largely involved in response to PS3 treatment. These results confirm the involvement of JA in PS3-IR, as previously demonstrated by Trouvelot et al. (2008). Besides, Gauthier et al. (2014) have reported that grapevine leaves treated by PS3 share a common stress-responsive transcriptome profile that partly overlaps the salicylate- (SA-) and JA-dependent one, although the microarray dataset was obtained at 12 hpt. The precise role of SA and JA in PS3-IR of grapevine against downy mildew thus remains to be elucidated.

Leaf samples were mock- or *P. viticola* inoculated at 48 hpt and collected for analysis at 72 and 96 hpt (corresponding to 24 and 48 hpi, respectively). As previously reported (Allègre et al., 2009), only PS3 IR against downy mildew (with mean values of disease severity of about 25 for W and H samples, and 4 for P ones at 144 hpt; data not shown). The global metabolic profile of mock-inoculated W samples did not change during the time course of experiment (data not shown), thus providing evidence for the “stability” of the global metabolism during the time course of the experiment. *P. viticola* infection clearly impacted the leaf metabolism. As it developed into W- and H-treated leaves, metabolites could also be the result of its own biosynthesis. However, samples were collected at the beginning of infection, when the biomass of the oomycete was low (Gamm et al., 2011), thus suggesting that it was rather due to plant response to infection. Ali et al. (2009) used NMR spectroscopy and (2D)-NMR techniques to obtain a metabolic profiling of susceptible *vs* resistant grapevine leaves upon inoculation with *P. viticola* at different time points. However, they did not provide a comparative global metabolite profile at 24 and 48 hpi, making it impossible to determine if a similar response was observed in their downy mildew susceptible variety.

All infected samples could also be discriminated at 72 and 96 hpt (72i and 96i samples) and presented fingerprints different to those observed at 48 hpt. Despite a time- and infection-dependent evolution, each sample set thus kept a specific metabolite fingerprint. Whatever the treatment, the number of top *m/z* decreased from 48 to 72 to 96 hpt, indicating that as time post treatment / post inoculation progressed, less masses marked the metabolic fingerprints. Interestingly, the weaker decrease of this number of top *m/z* was observed in response to PS3 treatment, i.e., in leaves where IR was effective.

H72i and H96i leaves did not present the same metabolic fingerprint as W72i and W96i ones, although *P. viticola* infection managed to develop inside them. These results show that despite H13 failed in inducing resistance, it nonetheless durably modulated the leaf metabolism. This is partly due to defense activation, as indicated by the occurrence of the phytoalexin resveratrol and derivatives, and also to the metabolic costs probably associated to this response.

The metabolic fingerprint of the three infected sample series changed upon time. The main pathways associated to each treatment and sampling time are summarized in **Table 3**. One of the main metabolic pathways associated to H72i and H96i was “secondary metabolism”, as for H48. The production of secondary metabolites was induced by H13 treatment and probably also by *P. viticola* infection. A greater number of metabolic pathways was associated to H48 and H96i, and included “carbon metabolism”, “carbon fixation in photosynthetic organism”, “pentose phosphate pathway”, and “biosynthesis of amino acid”. These results suggest a mobilization of the primary metabolism, with sugar and amino acid biosynthesis, and maybe photorespiration that represents a converging point for carbohydrate and amino acid metabolic pathways. These different pathways were reported for playing a role in plant defense responses (Rojas et al., 2014). They ensure the production of secondary metabolites and the associated energy costs. However, all these response failed to prevent the pathogen development. The “pentose glucuronate interconversions” pathway was mobilized in W72i, W96i and H96i samples and might highlight the plant response to *P. viticola* infection process. The metabolic response of P72i and P96i involved the “purine metabolism” pathway, as for P48, suggesting an active gene transcription and energy mobilization. The lipid metabolism was also involved in response to PS3 treatment, especially at 96 hpt, with masses annotated as linoleic acid, tetradecanoic acid, hexadecanoic acid. This means a possible membrane and cell wall rearrangement and the synthesis of fatty acid derivative compounds such as the signaling hormone JA which are key events associated to plant response to biotic cues (Rojas et al., 2014).

**Table 3:** Summary of the main metabolic pathways associated to annotated top *m/z* identified for each treatment and sampling time.

	W	H	P
48	Biosynthesis of secondary metabolites	**Biosynthesis of secondary metabolites Pentose and glucuronate interconversions Ascorbate and aldarate metabolism** Amino sugar and nucleotide sugar metabolism **Flavonoid biosynthesis** Starch and sucrose metabolism	Biosynthesis of secondary metabolites **Purine metabolism**
72i	**Pentose and glucuronate interconversions Ascorbate and aldarate metabolism**	**Biosynthesis of secondary metabolites Flavonoid biosynthesis**	Pyrimidine metabolism **Purine metabolism**
96i	**Pentose / glucuronate interconversions**	**Biosynthesis of secondary metabolites**	Linoleic acid metabolism


	**Ascorbate and aldarate metabolism**	Carbon fixation in photosynthetic organisms Carbon metabolism Biosynthesis of amino acids Pentose phosphate pathway **Pentose and glucuronate interconversions**	**Purine metabolism**

*In bold are highlighted the redundant ones. W, H, and P correspond to methanol extracts of grapevine leaf disks treated with H_*2*_O (W, as control), H (H13), or P (PS3) elicitor solutions (2.5 g.l^-*1*^) for 48 h*.

Metabolomics thereby provided us a better understanding of elicitor-induced resistance of grapevine leaves against *P. viticola*. Ballester et al. (2013) also used this approach to highlight the involvement of the phenylpropanoid pathway in the induction of resistance of Navelate oranges. Nevertheless, the use of metabolomics to investigate elicitor-induced resistance remains little documented. More studies have focused on plant interaction with microbes and pests (for review, see Balmer et al., 2013; Shiratake and Suzuki, 2016). As example, Hong et al. (2011) could discriminate healthy and botrytized berries of botrytized bunches, and berries of healthy bunches. All the analytical methods do not have the same performance and accuracy (Jorge et al., 2016; Maia et al., 2016; Shiratake and Suzuki, 2016) and plant/pathogen interactions have their specificity; making it difficult to compare the different results reported in the literature. However, a common observation revealed by metabolomic studies is the reprogramming of the plant metabolism, with an impact on both primary (often carbohydrates, amino acids) and secondary metabolites during interaction. This was described in barley, rice and *Brachypodium distachyon* in response to *Magnaporthe grisea* (Parker et al., 2009) or in rice in response to *Chilo suppressalis* attack (Liu et al., 2016). In grapevine, it was reported in *V. vinifera* cv. Trincadeira berries challenged by *Botrytis cinerea* (Agudelo-Romero et al., 2015) and leaves infected by *Plasmopara viticola* (Ali et al., 2009; Becker et al., 2013). Metabolomic also allows the identification of biomarkers of defensive or infected state. As example, Chin et al. (2014) reported biomarkers of citrus infection by the bacterium *Candidatus Liberibacter asiaticus*.

Putative erythritol phosphate (*m/z* = 201,01698, C_11_H_4_O_7_P) was the unique annotated compound specifically listed among the top *m/z* of PS3 treated samples. Little is known about the biological activity of this phosphorylated polyol. It was demonstrated that *Brucella* protobacteria can convert d-erythrose-4-phosphate to glyceraldehyde 3-phosphate and fructose 6-phosphate by enzymes of the pentose phosphate pathway, thus bypassing fructose-1,6-bisphosphatase (Barbier et al., 2014). In plants, its derivative methylerythritol phosphate was more studied as it constitutes an important pathway for the biosynthesis of isopentenyl diphosphate, a precursor of terpenes. As example, the synthesis of the diterpenoid phytoalexin in rice in response to elicitors (including JA) is mediated by this pathway (Okada et al., 2007). In grapevine, this pathway is also involved in the production of monoterpenes and sequiterpenes in response to methyljasmonate (Hampel et al., 2005). In this study, putative methylerythritol phosphate was present in all samples. Further studies will be necessary to confirm the identity of erythritol phosphate, to understand its regulation related to its methylated derivative, and to determine its role in PS3-IR. The reliability of this marker will have to be checked in other pathosystems and in response to other plant defense elicitors. Such a marker of elicitor-IR would be useful as a tool of selection for further screening of putative new elicitors. It would be also helpful to follow the plant response to elicitor treatment in field conditions and to study the impact of environmental conditions on elicitor-IR.

## Conclusion

The metabolomic approach used in this study allowed us to obtain specific and time-dependent metabolite signatures of PS3-IR, and also to identify a putative marker of PS3-IR. Despite *m/z* annotations and metabolic pathways could be associated to these signatures, this constitutes only partial information since numerous *m/z* remain “unknown” in databases. However, these results pave the way for future studies that will contribute to enrich our knowledge of elicitor-IR.

## Author Contributions

MA designed the work, conducted the experiments, contributed to data interpretation, and drafted the manuscript. BK performed FT-ICR-MS analysis. ML and CR-G performed data process and statistical analysis. BP, CL-G, M-CH, RG, PS-K, ST, and XD contributed to design the work and to interpret data. PS-K supervised the FT-ICR-MS analysis, and contributed to data interpretation. All authors reviewed, edited, and approved manuscript.

## Conflict of Interest Statement

The authors declare that the research was conducted in the absence of any commercial or financial relationships that could be construed as a potential conflict of interest.

## Footnotes

1 http://www.elicitra.org/index.php?vrs=english

## Supplementary Material

FIGURE S1 **Detailed visualization of all leaf samples analyzed by FT-ICR-MS**. The chemical histogram (left part) shows the distribution (number) of the elemental formulas attributed to the *m/z* obtained from all sample extracts according to their elemental composition (CHO, CHOS, CHON, CHONS, CHOP, CHONP, or CHONSP) and the Van Krevelen diagram (right part) represents these formulas onto two axes according to their H/C and O/C atomic ratios. Dots are colored according to their elemental composition (CHO, CHOS, CHON, CHONS, CHOP, CHONP, CHONSP) and sized according to their relative intensity in mass spectra.

FIGURE S2 **Detailed visualization of the 48 hpt samples analyzed by FT-ICR-MS**. The chemical histograms show the distribution (number) of the elemental formulas attributed to the *m/z* obtained from W48, H48, and P48 samples, respectively, according to their elemental composition (CHO, CHOS, CHON, CHONS, CHOP, CHONP, or CHONSP). The Van Krevelen diagrams represent these formulas onto two axes according to their H/C and O/C atomic ratios. Dots are colored according to their elemental composition (CHO, CHOS, CHON, CHONS, CHOP, CHONP, CHONSP) and sized according to their relative intensity in mass spectra. W48, H48, and P48 correspond to methanol extracts of grapevine leaf disks treated with H_2_O (W, as control), H (H13), or P (PS3) elicitor solutions (2.5 g.l^-1^) for 48 h.

FIGURE S3 **Discrimination of the 72 and 96i samples from FT-ICR-MS data**. Venn diagrams showing the distribution of the total **(A,C)** and top **(B,D)** *m/z* obtained by (–) FT-ICR-MS analysis of W, H, P infected samples collected at 72 and 96 hpt, respectively. W, H, and P correspond to methanol extracts of grapevine leaf disks treated with H_2_O (W, as control), H (H13), or P (PS3) elicitor solutions (2.5 g.l^-1^) for 48 h and inoculated with *P. viticola*. Top *m/z* correspond to *m/z* with the highest regression coefficient value (VIP ≥ 1).

FIGURE S4 **Detailed visualization of the 72i and 96i samples analyzed by FT-ICR-MS**. The Van Krevelen diagrams represent the elemental formulas attributed to the top *m/z* of W72i **(A)**, H72i **(B)**, P72i **(C)**, W96i **(D)**, H96i **(E)**, and P96i **(F)** samples onto two axes according to their H/C and O/C atomic ratios. Dots are colored according to their elemental composition (CHO, CHOS, CHON, CHONS, CHOP, CHONP, CHONSP) and sized according to their relative intensity in mass spectra. Diagrams (in the bottom left corner) show the distribution of these formulas according to their elemental composition (CHO, CHOS, CHON, CHONS, CHOP, CHONP, or CHONSP). W72i/96i, H72i/96i, and P72i/96i correspond to methanol extracts of grapevine leaf disks treated with distilled H_2_O (W, as control), H (H13), or P (PS3) elicitor solutions (2.5 g.l^-1^) for 48 h and collected 72 and 96 h after *P. viticola* inoculation. Top *m/z* correspond to *m/z* with the highest regression coefficient value (VIP ≥ 1).

TABLE S1 **Annotations of W48, H48 and P48 top *m/z* after query of KEGG databasis (*Vitis vinifera* organism) with the MassTRIX interface**.

TABLE S2 **Annotations of 72i and 96i top *m/z* for W, H, and P series after query of KEGG databasis (*V. vinifera* organism) with the MassTRIX interface**.

TABLE S3 **list of the transformations targeted on erythritol phosphate metabolism**.
