# Supplementary figures and images for: Metabolic Fingerprint of PS3-Induced Resistance of Grapevine Leaves against Plasmopara viticola Revealed Differences in Elicitor-Triggered Defenses
Source: Front Plant Sci. 2017 Feb 14;8:101. doi: 10.3389/fpls.2017.00101 (PMC5306141; doi:10.3389/fpls.2017.00101)

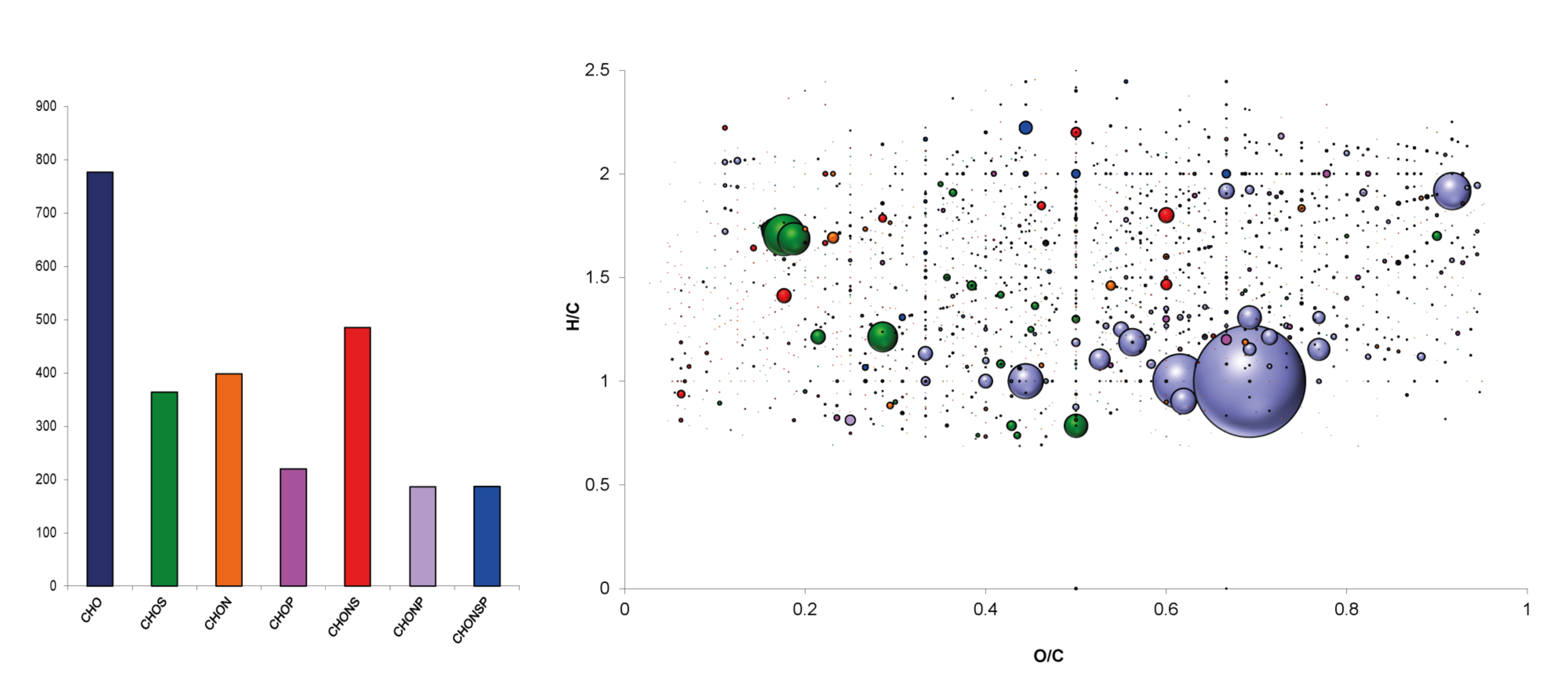

Supplement: FIGURE S1 — Detailed visualization of all leaf samples analyzed by FT-ICR-MS. The chemical histogram (left part) shows the distribution (number) of the elemental formulas attributed to the m/z obtained from all sample extracts according to their elemental composition (CHO, CHOS, CHON, CHONS, CHOP, CHONP, or CHONSP) and the Van Krevelen diagram (right part) represents these formulas onto two axes according to their H/C and O/C atomic ratios. Dots are colored according to their elemental composition (CHO, CHOS, CHON, CHONS, CHOP, CHONP, CHONSP) and sized according to their relative intensity in mass spectra. [file Image_1.TIF]

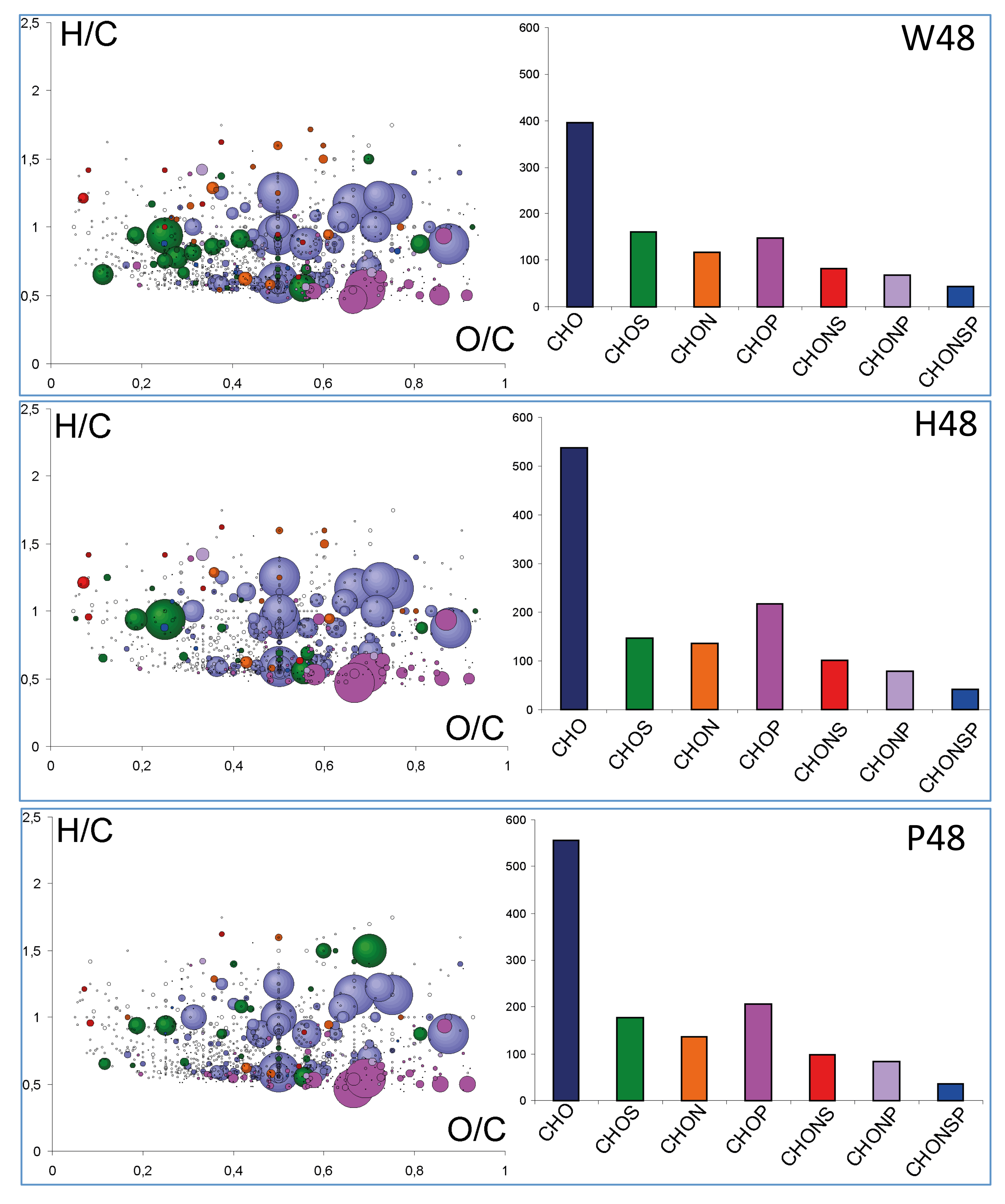

Supplement: FIGURE S2 — Detailed visualization of the 48 hpt samples analyzed by FT-ICR-MS. The chemical histograms show the distribution (number) of the elemental formulas attributed to the m/z obtained from W48, H48, and P48 samples, respectively, according to their elemental composition (CHO, CHOS, CHON, CHONS, CHOP, CHONP, or CHONSP). The Van Krevelen diagrams represent these formulas onto two axes according to their H/C and O/C atomic ratios. Dots are colored according to their elemental composition (CHO, CHOS, CHON, CHONS, CHOP, CHONP, CHONSP) and sized according to their relative intensity in mass spectra. W48, H48, and P48 correspond to methanol extracts of grapevine leaf disks treated with H2O (W, as control), H (H13), or P (PS3) elicitor solutions (2.5 g.l-1) for 48 h. [file Image_2.TIF]

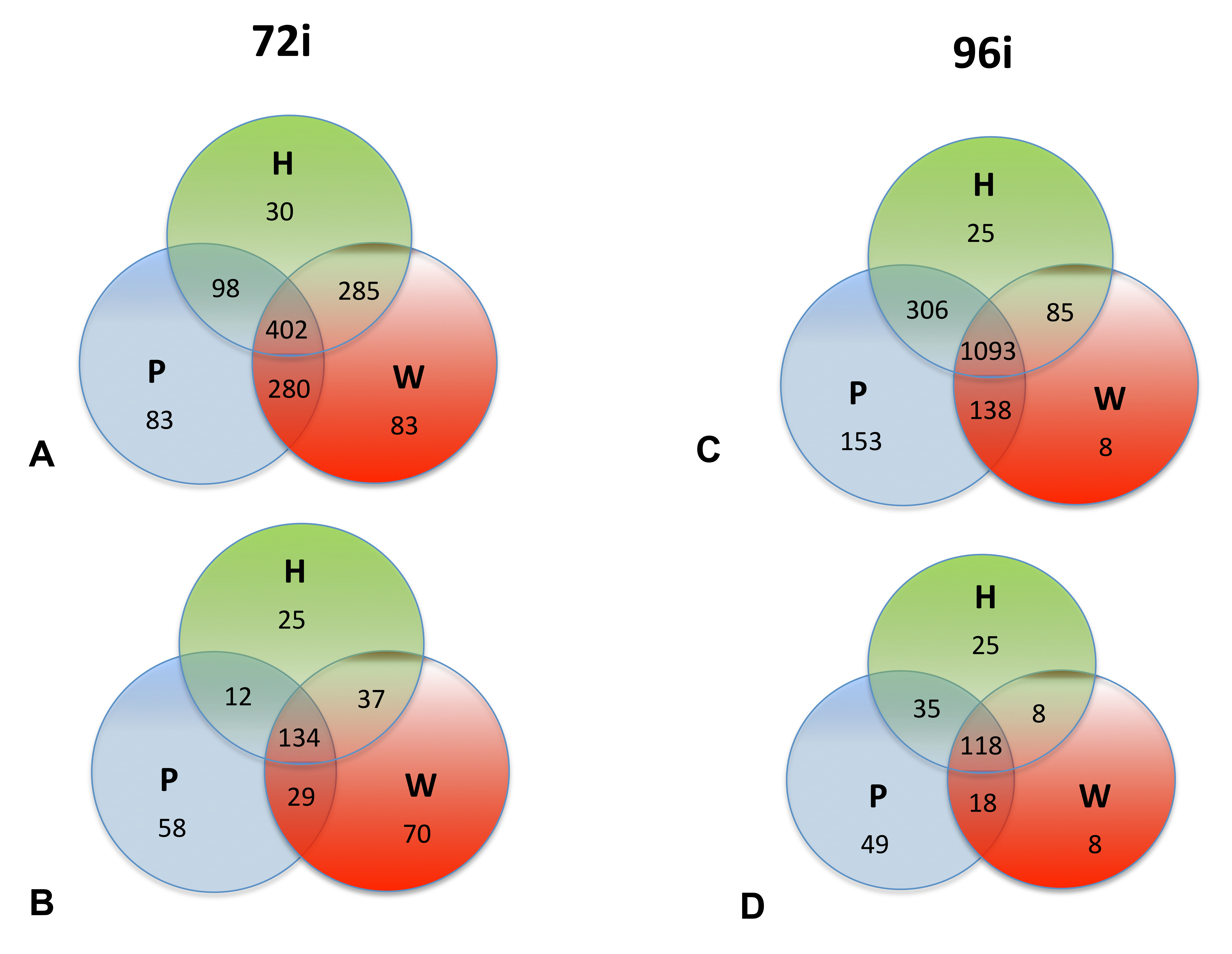

Supplement: FIGURE S3 — Discrimination of the 72 and 96i samples from FT-ICR-MS data. Venn diagrams showing the distribution of the total (A,C) and top (B,D) m/z obtained by (–) FT-ICR-MS analysis of W, H, P infected samples collected at 72 and 96 hpt, respectively. W, H, and P correspond to methanol extracts of grapevine leaf disks treated with H2O (W, as control), H (H13), or P (PS3) elicitor solutions (2.5 g.l-1) for 48 h and inoculated with P. viticola. Top m/z correspond to m/z with the highest regression coefficient value (VIP ≥ 1). [file Image_3.TIF]

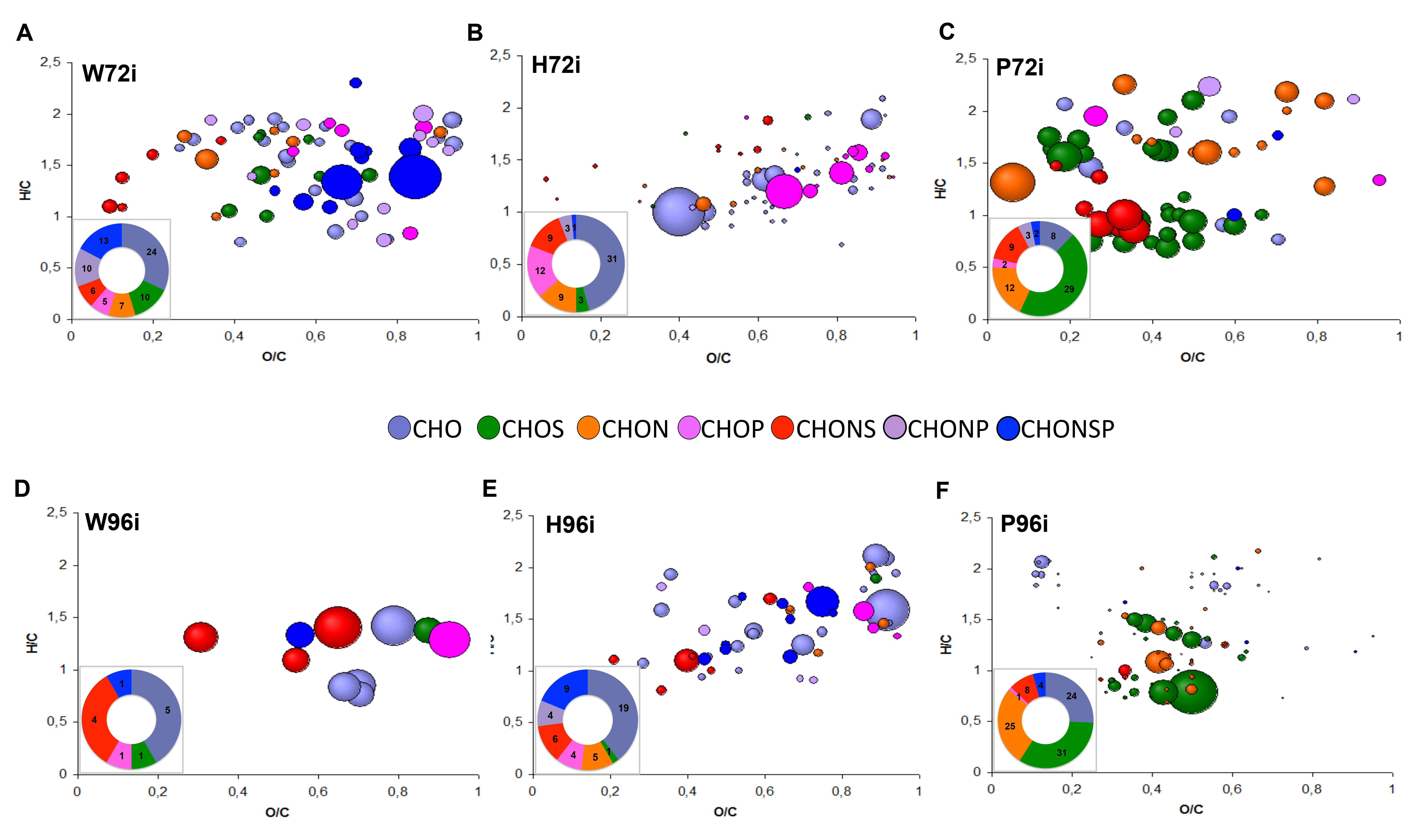

Supplement: FIGURE S4 — Detailed visualization of the 72i and 96i samples analyzed by FT-ICR-MS. The Van Krevelen diagrams represent the elemental formulas attributed to the top m/z of W72i (A), H72i (B), P72i (C), W96i (D), H96i (E), and P96i (F) samples onto two axes according to their H/C and O/C atomic ratios. Dots are colored according to their elemental composition (CHO, CHOS, CHON, CHONS, CHOP, CHONP, CHONSP) and sized according to their relative intensity in mass spectra. Diagrams (in the bottom left corner) show the distribution of these formulas according to their elemental composition (CHO, CHOS, CHON, CHONS, CHOP, CHONP, or CHONSP). W72i/96i, H72i/96i, and P72i/96i correspond to methanol extracts of grapevine leaf disks treated with distilled H2O (W, as control), H (H13), or P (PS3) elicitor solutions (2.5 g.l-1) for 48 h and collected 72 and 96 h after P. viticola inoculation. Top m/z correspond to m/z with the highest regression coefficient value (VIP ≥ 1). [file Image_4.TIF]
